# The role of implants and implant prostheses on the accuracy and artifacts of cone-beam computed tomography: an in-vitro study

**DOI:** 10.1038/s41598-024-51293-3

**Published:** 2024-01-06

**Authors:** Balwant Singh Gurjar, Vineet Sharma, Jyoti Paliwal, Rajani Kalla, Kamal Kumar Meena, Mohammed Tahir

**Affiliations:** Department of Prosthodontics, RUHS College of Dental Sciences, Jaipur, India

**Keywords:** Dentistry, Dental radiology, Prosthetic dentistry

## Abstract

To assess the accuracy of CBCT in implant-supported prostheses and to evaluate metal artifacts with and without implants or implant prostheses. Accuracy and artifacts were assessed in the dried mandible at three points on the buccal and lingual cortical plates on the mandible's body near the crest and the base. On the buccal cortical plate, these points were labelled as A, B and C near the crest and D, E and F near the base of the body of the mandible. Similarly, points a to f were marked on the lingual cortical plate corresponding to points A to F. The study had two control groups, C0 for physical linear measurement (PLM) and C1 for radiographic linear measurement (RLM) and artifact assessment. There were seven test groups, TG 1 to 7, progressing from a single implant to implant full-arch prosthesis. For accuracy assessment, PLM was compared to RLM. CBCT artifacts were investigated in images integrated at 0.25 mm, 10 mm, and 20 mm at regions of interest on concentric circles at different intersecting angles by comparing grayscale values at C1 and TG1 to 7. The data were collected and statistically analyzed. A significant difference was observed between C0 and C1, and RLM in test groups at the superior axial plane. Similarly, PLM and test RLM in the sagittal plane at A-B, B-C, and D-E were statistically significant. A significant difference between PLM and RLM was also observed in the vertical plane at A-D, B-E, and C-F. Quantification of CBCT artifacts in the presence of implants or prostheses revealed that full-arch prostheses had the highest mean grayscale value, whereas single implants with a prosthesis had the lowest. The mean grayscale change was greatest around the implant and implant prosthesis. The mean grayscale value was maximum at 20 mm voxel integration scales (VIS) and lowest at 0.25 mm. CBCT is a clinically reliable device. Metal in implants or implant-supported prostheses prevents true assessment of the peri-implant area; therefore, lower VIS is suggested in the presence of implants or implant prostheses.

## Introduction

Radiological imaging has become increasingly important for diagnosis, clinical assessment, and treatment planning^1^. In recent years, technology has advanced from two-dimensional conventional radiographs to three-dimensional digital imaging such as computed tomography (CT), magnetic resonance imaging (MRI), and cone-beam computed tomography (CBCT)^2^.

CBCT is now an important part of diagnosis and treatment in fields like maxillofacial surgery, orthodontics, endodontics, implant planning, and evaluating patients after treatment^3–7^. Nevertheless, the presence of metals or alloys in implants, posts, cores, crowns, bridges, and amalgam fillings decreases image quality in the area adjacent to them due to reduced contrast caused by beam hardening, scattering effect, partial volume or edge effect, aliasing artifacts, and ring artifacts^8–10^. Due to increased life expectancy and the consequent increase in partial or complete edentulism, implant-supported prostheses have also grown in popularity^10^.

In their studies, Torres^11^ and Sheikhi^12^ found the physical linear measurements to be more than radiographic CBCT measurements. Linear measurements of alveolar bone and maxillofacial structures by CBCT were proven accurate, trustworthy, and independent of the presence of dental implants by Ekrish^13^ and Amarnath^14^. Beam hardening artifacts were observed in the presence of metal implants in studies conducted by Schulze^15^ and Dreanert^16^.

The current study sought to evaluate the accuracy of CBCT as a measuring tool and quantify the artifact, if any, in the presence of implants and implant-supported prostheses by measuring and comparing linear distances in transverse, sagittal, and vertical planes in the physical model to radiographic measurements, as well as evaluating metal artifacts produced by CBCT of the mandible with or without implants or prostheses.

The null hypothesis stated that the accuracy of CBCT images is unaffected by the presence of implants and implant-supported prostheses.

## Materials and methods

**Figure 1:**
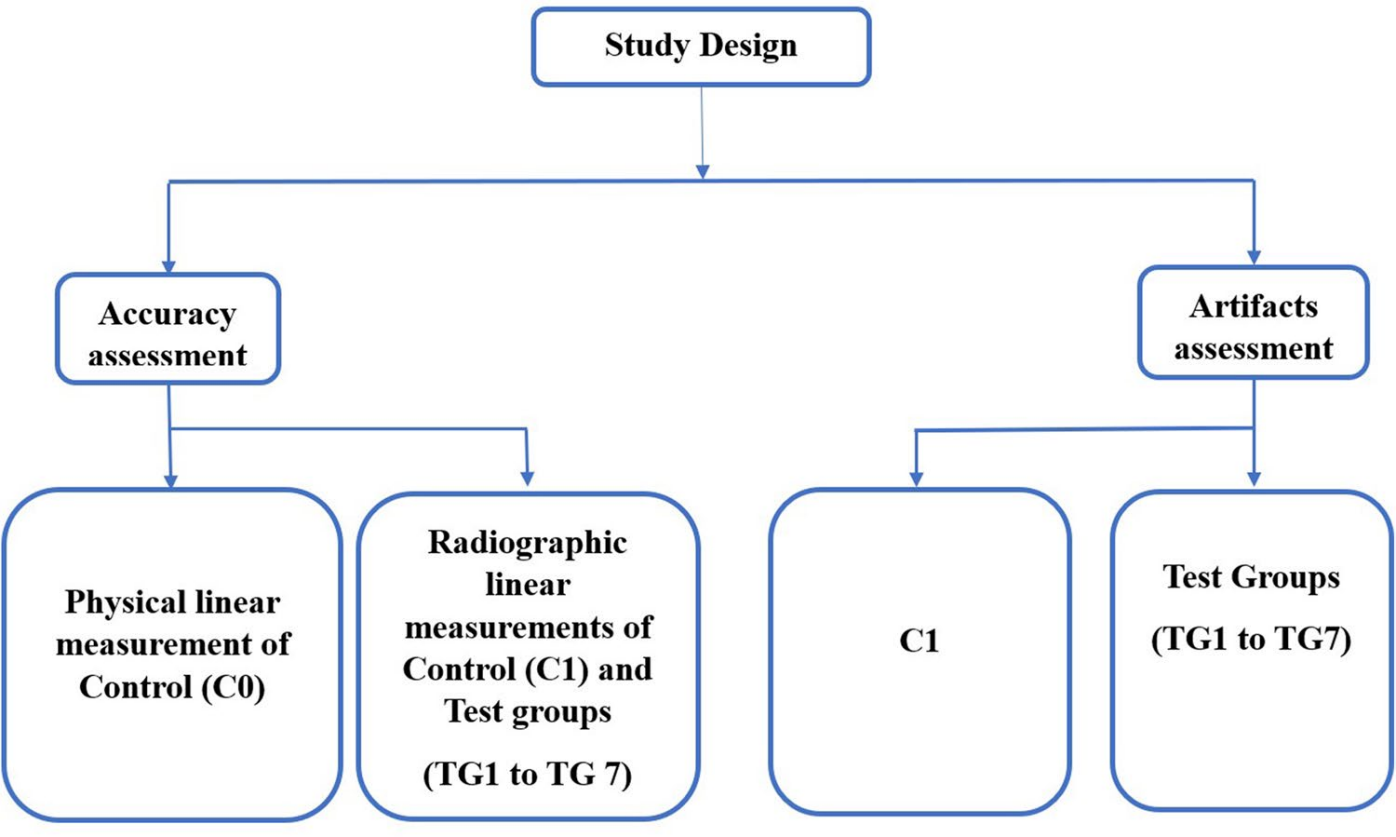
Flow-chart depicting the study design for assessment of dimensional accuracy and artifacts in CBCT at different stages. C0 = Absolute Control, C1 = Radiographic Control, TG1 = Single Implant placed at 43, TG2 = Implant-supported Porcelain fused to metal (PFM) crown with the abutment at 43, TG3 = Two Implants placed at 43 and 46, TG4 = Two Implant-supported PFM crowns at 43 and 46, TG5 = Implant-supported four-unit PFM prosthesis from 43 to 46, TG6 = Implant-supported four-unit PFM prostheses from 43 to 46 and 33 to 36 (Implants placed at 43, 46, 33 and 36), TG7 = Full arch implant-supported prosthesis.

**Figure 2:**
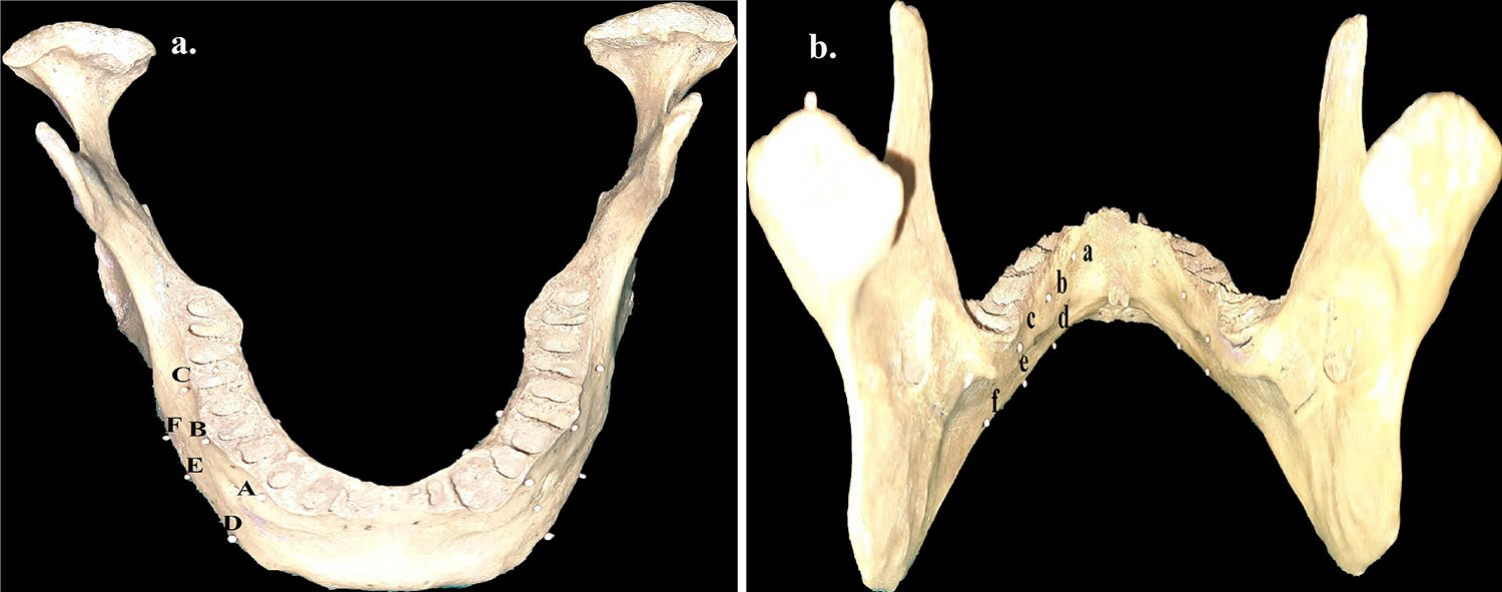
Reference points for accuracy measurement. (**a**) Buccal reference points for accuracy measurement at the crest of the mandible (A, B, C) and the base of the mandible (D, E, F). (**b**) Lingual reference points for accuracy measurement at the crest of the mandible (a, b, c) and the base of the mandible (d, e, f).

The research was conducted at the RUHS College of Dental Sciences in Jaipur, in the Department of Prosthodontics. The study received ethical committee clearance no. EC/PG-015/2019. The study used dry edentulous mandible and endosteal tapered SLA-treated (4 × 10 mm) titanium implants with titanium abutments (Superline Dentium implant system; Dentium South Korea). For assessment of accuracy, two points were marked on the buccal cortical plate of the body of a dried edentulous mandible, corresponding to the right mandibular canine (A and D) and mandibular first molar (C and F), both near the crest and base, respectively. Third point B and E were subsequently marked between points A and C and D and F respectively. Similarly, points a to f, corresponding to points A to F on the buccal cortical plate, were marked on the lingual cortical plate. This was referred to as ‘Absolute Control (C0)’ and was used for physical linear measurement (PLM). The radio-opaque gutta-percha balls of 0.5 mm diameter were then affixed using cyanoacrylate adhesive through points A to F and a to f on the buccal and lingual plates, respectively. This was referred to as radiographic control (C1), for radiographic linear measurements (RLM) in different spatial configurations. These reference points provide a standardized and reproducible framework for measuring and comparing PLM with RLM. The specific locations of the points were chosen to provide a reference for measurements in the transverse, sagittal, and vertical planes, which would be important for assessing the accuracy of CBCT images in the study (Figs. 1, 2).

**Figure 3:**
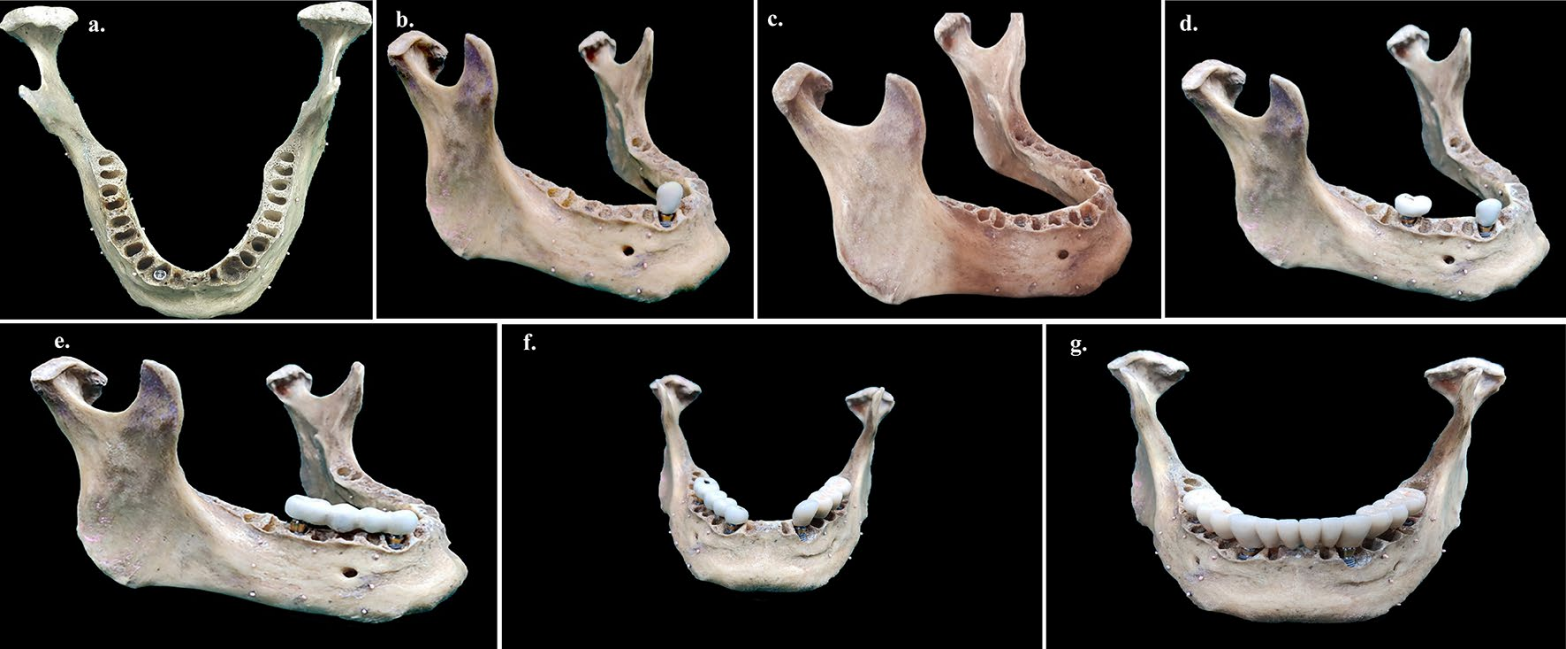
Stages of study model. (**a**) 4 × 10 mm titanium implant placed at 43 (TG1). (**b**) Implant-supported PFM crown with the abutment at 43 (TG2). (**c**) 4 × 10 mm titanium implants placed in the study model at 43 and 46 (TG3). (**d**) Implant-supported PFM single crowns at 43 and 46 (TG4). (**e**) Implant-supported four-unit PFM prosthesis from 43 to 46 (TG5). (**f**) Implant-supported four-unit PFM prostheses from 43 to 46 and 33 to 36 with 4 × 10 mm titanium implants placed in canine and first molar region bilaterally at 33, 43, 36, and 46 (TG6). (**g**) Full-arch implant-supported prosthesis (TG7).

Furthermore, seven test groups (TG1 to TG7) were created (Fig. 3). A styrofoam customized base was used to standardize the position of the mandible, with the base parallel to the horizontal plane and centered within the CBCT field of view (FOV). CBCT images were acquired using a CBCT machine (Care Stream CS3D-9000 machine; Care Stream Health Inc). The image volume was reconstructed with isotropic isometric 250 × 250 × 250 μm voxels after receiving a scout image to include the region of interest.

Three independent observers performed PLM with a digital vernier caliper with a sensitivity of 0.01 mm. To determine dimensional accuracy, PLM was compared to RLM in all three planes: transverse, sagittal, and vertical.

RLM were performed in multi-planar oblique CBCT sections using the CBCT software measurement tool (Carestream 3D imaging, version 3.10.8; Carestream Health, Inc). RLM was performed on radiographic points in the transverse, sagittal, and vertical planes corresponding to physical points.

**Figure 4:**
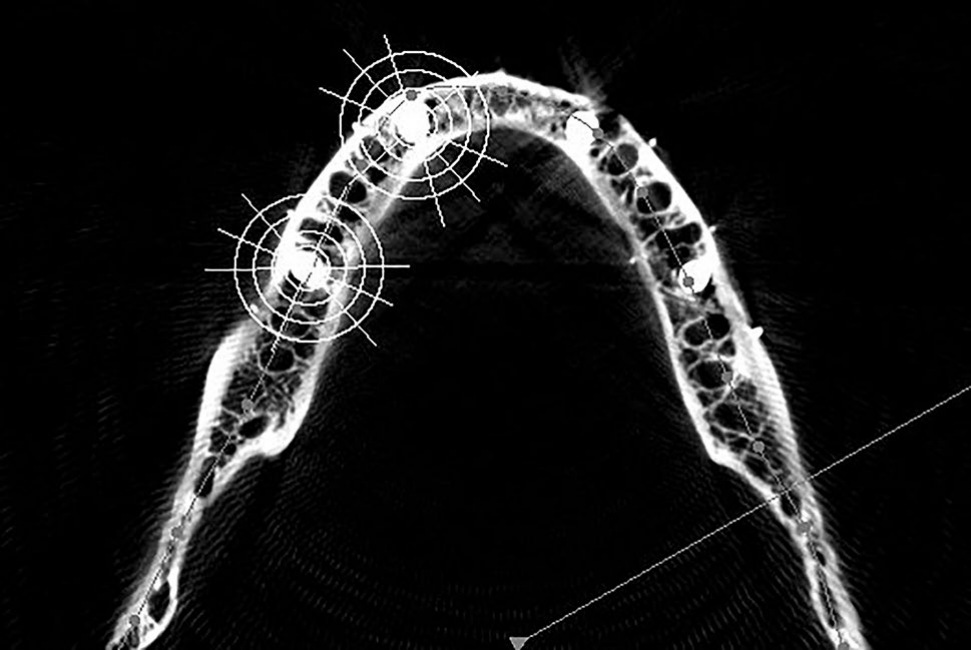
CBCT image depicting the region of interest for implant at canine and molar region.

**Figure 5:**
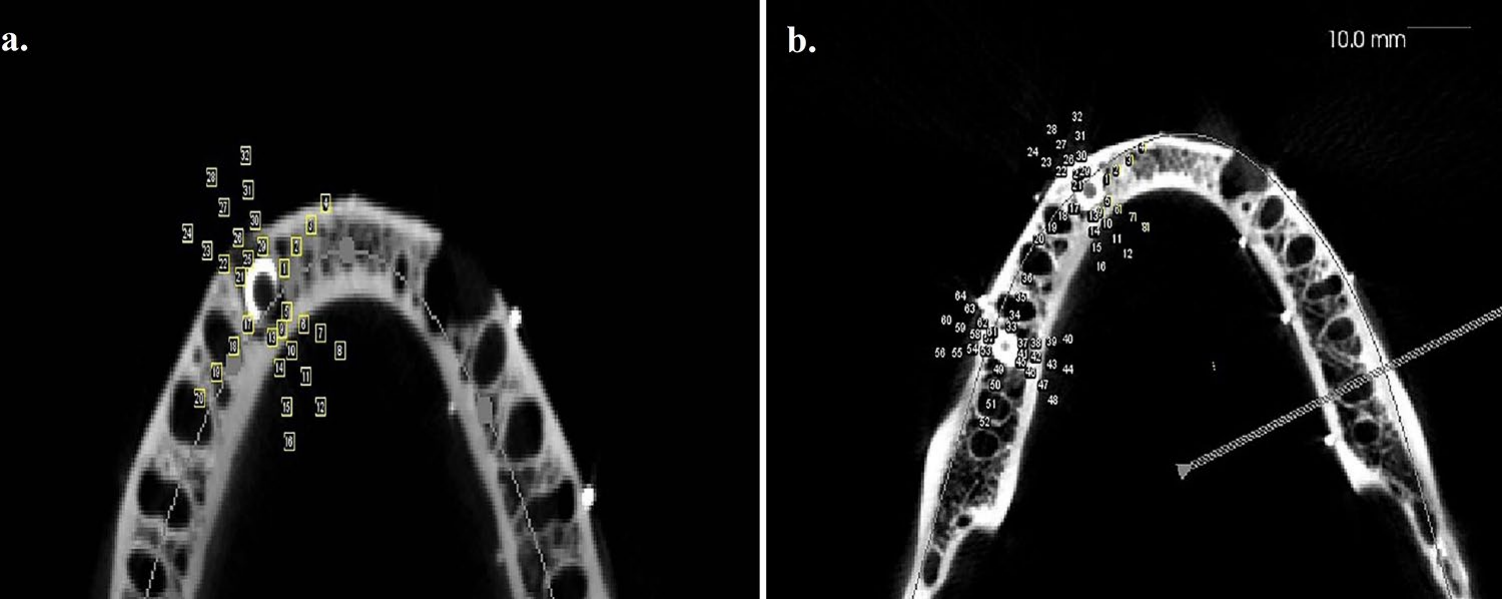
CBCT image depicting points ROI for the implant. (**a**) At canine (1–32). (**b**) At canine and molar region (1–64).

**Figure 6:**
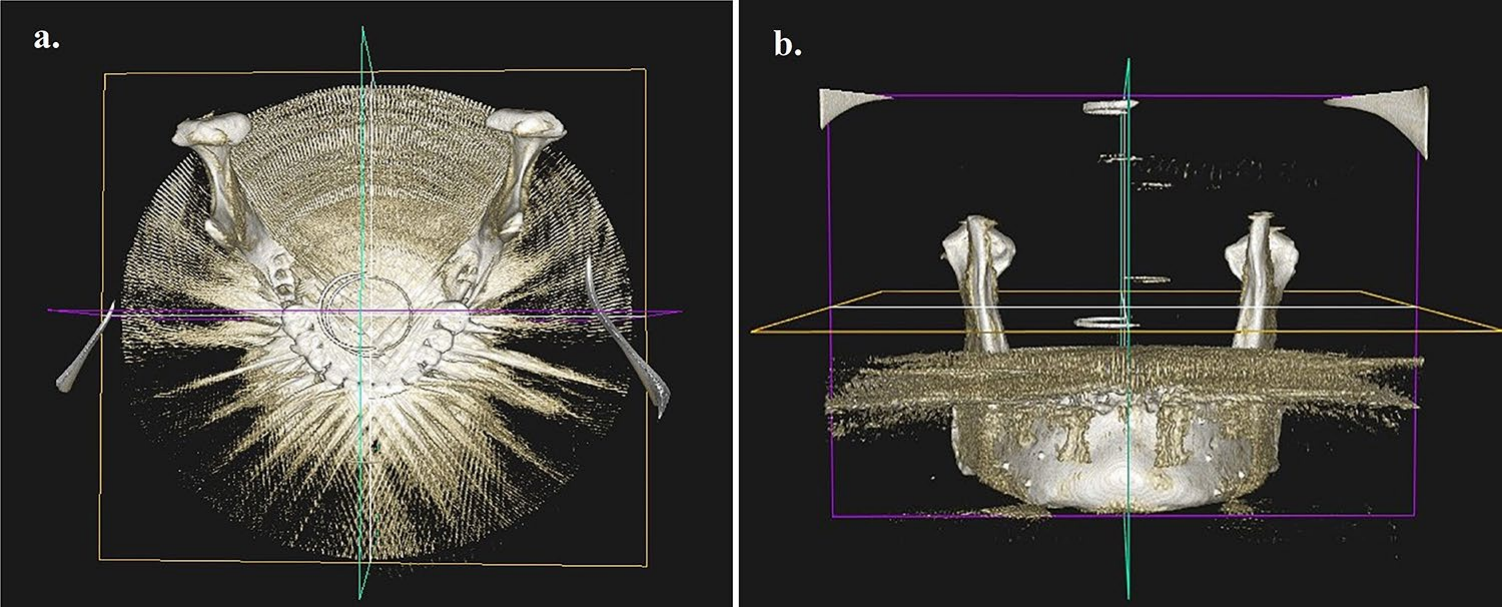
3-D CBCT image showing artifacts with implants at 33, 36, 43, and 46 and full arch prosthesis. (**a**) Occlusal view. (**b**) Frontal view.

The artifacts were examined at locations A, B, a, and b in the superior axial plane. The images were integrated at a scale of 0.25 mm, 10.8 mm, and 21.8 mm and saved using the CBCT workplace screenshot tool. The screenshot was transformed to a 16-bit grayscale image using image software (The image J program, version 1.530; National Institute of Health and the Laboratory for Optical and Computational Instrumentation (LOCI)—University of Wisconsin) (grayscale range 0–255). Concentric circles with diameters of 6 mm, 10 mm, 15 mm, and 20 mm were drawn around the center of the implant at the canine and molar regions to define the region of interest. A reference line (Ro) was drawn across the center of the implant and parallel to the mandible's buccal and lingual cortical plates. Intersecting lines were made with respect to Ro at 0°, 65°, 90°, 115°,180°, 245°, 270°, and 295°, intersecting the concentric circles to yield points of interest (Fig. 4). Macro (macro record tool of Image-J software version 1.530, National Institute of Health and the Laboratory for Optical and Computational Instrumentation (LOCI), University of Wisconsin) was used to delineate 32, 1-mm square regions of interest (ROI) for both implants placed at 43 and 46. Finally, the recorded Macro was run on an unmarked image to capture the average grayscale values of the square ROI mentioned previously (Figs. 5, 6). The artifacts in CBCT were recorded in the study at C1 and TG1 through TG7. The artifacts of the superior axial plane were then compared to those of the inferior axial plane (which corresponded to the appearance of the superior border of the mental foramen).

For osteotomy, conventional implant drilling protocols were followed for the Dentium surgical kit (SuperLine & Implantium Surgical Kit; Dentium). All Porcelain fused to metal (PFM) prostheses were fabricated by the lost wax technique using a base metal casting alloy.

The data obtained was compiled on an MS Office Excel sheet (v. 2016). The Statistical Package for the Social Sciences (SPSS) version 21.0 (IBM) was used to analyze the data. A one-way ANOVA test was used for intergroup comparison. The intra-group comparison was made using the post-hoc Tukey test. For all the statistical tests, p < 0.05 was considered statistically significant, keeping α error at 5% and giving 95% power to the study.

## Results

The dimensional accuracy of CBCT as a measuring instrument was determined by comparing the RLM of C1 with the PLM (C0) in the transverse, sagittal, and vertical planes. The observed p-values after the paired t-test were statistically non-significant (p > 0.05) in all three planes.

In the transverse plane, the PLM (C0) was 9.23 mm, 10.77 mm, and 10.63 mm for A-a, B-b, and C–c in the superior plane, while the minimum and maximum RLM for test groups observed were 9.37 mm (TG1) and 9.63 mm (TG7), 10.57 mm (TG1) and 10.93 mm (TG6), and 9.37 mm (TG4 and TG5) and 10.73 mm (TG6) for A-a, B-b, and C-c, respectively. Similarly, PLM(C0) for D-d, E-e, and F-f in the inferior plane was 8.30 mm, 8.12 mm, and 7.19 mm, respectively, as compared to the observed minimum and maximum RLM values of 9.37 mm (TG1, TG4, and TG5) and 8.37 mm (TG7), 7.83 mm (TG5) and 8.03 mm (TG1 and TG7), 6.83 mm (TG7) and 7.13 mm (TG4 and TG5) for D-d, E-e, F-f thus, showing statistically significant results in the superior axial plane (Table 1).

**Table 1:** Comparative assessment in transverse plane: A one-way ANOVA comparison followed by post-hoc Tukey HSD test.

Test groups	N	Mean value (mm)	Standard deviation	Mean difference	Standard error	95% confidence interval		Significance
						Lower bound	Upper bound	
A-a								
C0	3	9.23^ab^	0.060					
C1	3	9.37	0.060	− 0.1333	0.0875	− 0.44	0.173	0.009*
TG1	3	9.37	0.060	− 0.1333	0.0875	− 0.44	0.173	
TG2	3	9.50	0.100	− 0.2667	0.0875	− 0.573	0.04	
TG3	3	9.37	0.060	− 0.1333	0.0875	− 0.44	0.173	
TG4	3	9.40	0.100	− 0.1667	0.0875	− 0.473	0.14	
TG5	3	9.57^a^	0.210	− 0.3333*	0.0875	− 0.64	− 0.027	
TG6	3	9.43	0.060	− 0.2	0.0875	− 0.507	0.107	
TG7	3	9.63^b^	0.150	− .4000*	0.0875	− 0.707	− 0.093	
B-b								
C0	3	10.77^a^	10.770					0.000*
C1	3	10.78^b^	10.780	− 0.00667	0.04869	− 0.1773	0.1639	
TG1	3	10.57^abcdef^	10.570	0.20333*	0.04869	0.0327	0.3739	
TG2	3	10.63^ghij^	10.630	0.13667	0.04869	− 0.0339	0.3073	
TG3	3	10.83^cg^	10.830	− 0.06333	0.04869	− 0.2339	0.1073	
TG4	3	10.70^kl^	10.700	0.07	0.04869	− 0.1006	0.2406	
TG5	3	10.87^dh^	10.870	− 0.09667	0.04869	− 0.2673	0.0739	
TG6	3	10.93^eik^	10.930	− 0.16333	0.04869	− 0.3339	0.0073	
TG7	3	10.90^fjl^	10.9	− 0.13	0.04869	− 0.3006	0.0406	
C-c								
C0	3	10.63	10.630					0.004
C1	3	10.63	10.630	0	0.03852	− 0.135	0.135	
TG1	3	10.67	10.670	− 0.03333	0.03852	− 0.1683	0.1016	
TG2	3	10.60	10.600	0.03333	0.03852	− 0.1016	0.1683	
TG3	3	10.67	10.670	− 0.03333	0.03852	− 0.1683	0.1016	
TG4	3	10.57^ab^	10.570	0.06667	0.03852	− 0.0683	0.2016	
TG5	3	10.57^a^	10.570	0.06667	0.03852	− 0.0683	0.2016	
TG6	3	10.73^b^	10.730	− 0.1	0.03852	− 0.235	0.035	
TG7	3	10.63	10.700	− 0.06667	0.03852	− 0.2016	0.0683	
D-d								
C0	3	8.300	0.020					0.356
C1	3	8.230	0.060	0.06667	0.11235	− 0.327	0.4603	
TG1	3	8.130	0.060	0.16667	0.11235	− 0.227	0.5603	
TG2	3	8.200	0.100	0.1	0.11235	− 0.2937	0.4937	
TG3	3	8.170	0.060	0.13333	0.11235	− 0.2603	0.527	
TG4	3	8.130	0.060	0.16667	0.11235	− 0.227	0.5603	
TG5	3	8.130	0.060	0.16667	0.11235	− 0.227	0.5603	
TG6	3	8.300	0.000	0	0.11235	− 0.3937	0.3937	
TG7	3	8.370	0.380	− 0.06667	0.11235	− 0.4603	0.327	
E-e								
C0	3	8.12	0.03					0.051
C1	3	8.07	0.06	0.05333	0.0743	− 0.207	0.3137	
TG1	3	8.03	0.06	0.08667	0.0743	− 0.1737	0.347	
TG2	3	7.93	0.06	0.18667	0.0743	− 0.0737	0.447	
TG3	3	7.97	0.06	0.15333	0.0743	− 0.107	0.4137	
TG4	3	7.97	0.06	0.15333	0.0743	− 0.107	0.4137	
TG5	3	7.83	0.21	0.28667*	0.0743	0.0263	0.547	
TG6	3	8	0.1	0.12	0.0743	− 0.1403	0.3803	
TG7	3	8.03	0.06	0.08667	0.0743	− 0.1737	0.347	
F-f								
C0	3	7.19^abcd^	0.02					0.052
C1	3	7.17^efgh^	0.12	0.02667	0.05009	− 0.1488	0.2022	
TG1	3	7.07^i^	0.06	0.12667	0.05009	− 0.0488	0.3022	
TG2	3	6.97^ae^	0.06	0.22667*	0.05009	0.0512	0.4022	
TG3	3	6.93^bfjk^	0.06	0.26000*	0.05009	0.0845	0.4355	
TG4	3	7.13^jlm^	0.06	0.06	0.05009	− 0.1155	0.2355	
TG5	3	7.13^kno^	0.06	0.06	0.05009	− 0.1155	0.2355	
TG6	3	6.90^cgln^	0	0.29333*	0.05009	0.1178	0.4688	
TG7	3	6.83^dhimo^	0.06	0.36000*	0.05009	0.1845	0.5355	

*p* > 0.05 statistically non-significant difference.

* -p < 0.05-statistically significant difference.

The identical superscript indicates statistically significant difference amongst designated groups for respective linear measurement (eg. A-a, B-b…F-f).

The sagittal plane PLM for A-B, B-C, D-E, and E-F was 15.47 mm, 12.25 mm, 15.72 mm, and 10.83 mm, respectively, with minimum and maximum test group RLM values of 15.53 mm (TG4, TG6, and TG7) and 15.93 mm (TG5), 12.13 mm (TG4) and 12.27 mm (TG2), 16.03 mm (TG6) and 16.27 mm (TG3), 10.83 mm (TG6 and TG7) and10.97 mm (TG1) for A-B, B-C, D-E, E-F. PLM and test RLM at A-B, B-C, and D-E in the sagittal plane were statistically significant (p < 0.01) (Table 2).

**Table 2:** Comparative assessment in the sagittal plane: using one-way ANOVA test.

Test groups	Mean	Standard deviation	F value	p-value
A-B				
C0	15.47^a^	0.12	17.443	0.000*
C1	15.13^bcdefgh^	0.15		
TG1	15.70^ab^	0.10		
TG2	15.55^ci^	0.13		
TG3	15.63^dj^	0.06		
TG4	15.53^ek^	0.06		
TG5	15.93^afijkgh^	0.06		
TG6	15.53^g^	0.06		
TG7	15.53^h^	0.06		
B-C				
C0	12.25	0.05	3.984	0.007*
C1	12.41^ab^	0.10		
TG1	12.30	0.10		
TG2	12.27	0.06		
TG3	12.17^a^	0.06		
TG4	12.13^b^	0.06		
TG5	12.23	0.06		
TG6	12.23	0.06		
TG7	12.23	0.06		
D-E				
C0	15.72^abcdefgh^	0.08	6.695	0.000*
C1	16.16^a^	0.26		
TG1	16.23^b^	0.06		
TG2	16.25^c^	0.05		
TG3	16.27^d^	0.06		
TG4	16.23^e^	0.06		
TG5	16.27^f^	0.06		
TG6	16.03^g^	0.06		
TG7	16.07^h^	0.06		
E-F				
C0	10.83	0.06	2.452	0.054
C1	10.82	0.07		
TG1	10.97	0.06		
TG2	10.93	0.06		
TG3	10.90	0.00		
TG4	10.87	0.06		
TG5	10.87	0.06		
TG6	10.83	0.06		
TG7	10.83	0.06		

*p* > 0.05 statistically non-significant difference.

* -p < 0.05-statistically significant difference.

The identical superscript indicates statistically significant difference amongst designated groups for respective linear measurement (eg. A-B, B-C…E–F).

The vertical plane PLM (C0) for A-D, B-E, and C-F was 15.43 mm, 14.36 mm, and 15.35 mm, respectively, with a minimum and maximum value of test group RLM observed of 15.23 mm (TG1) and 15.63 mm (TG4, TG5, and TG7), 13.37 mm (TG6) and 14.33 mm (TG2, TG4, and TG5), 14.93 mm (TG4 and TG5), and 15.47 mm (TG1), respectively, for A-D, B-E, and C-F. There was a statistically significant difference between PLM and RLM at A-D, B-E, and C-F (p < 0.01) (Table 3). An intra-group analysis of test CBCT also revealed statistically significant differences across groups.

**Table 3:** Comparative assessment in Vertical plane: using one-way ANOVA test.

Groups	Mean measurements (mm)	Standard deviation	p-value
A-D			
C0	15.43	0.15	0.003*
C1	15.43	0.06	
TG1	15.23^abc^	0.06	
TG2	15.43	0.21	
TG3	15.53	0.12	
TG4	15.63^a^	0.06	
TG5	15.63^b^	0.06	
TG6	15.53	0.06	
TG7	15.63^c^	0.06	
B-E			
C0	14.36^a^	0.08	0.000*
C1	14.40^b^	0.10	
TG1	14.23^c^	0.06	
TG2	14.33^d^	0.06	
TG3	14.27^e^	0.06	
TG4	14.33^f^	0.06	
TG5	14.33^g^	0.06	
TG6	13.37^abcdefg^	0.12	
TG7	13.90^abcdefg^	0.00	
C-F			
C0	15.35^ai^	0.06	0.000*
C1	15.45^begj^	0.17	
TG1	15.47^cfhk^	0.12	
TG2	15.37^dl^	0.06	
TG3	15.17	0.15	
TG4	14.93^abcd^	0.06	
TG5	14.93^ijkl^	0.06	
TG6	15.13^ef^	0.06	
TG7	15.13^gh^	0.06	

*p* > 0.05 statistically non-significant difference.

* -p < 0.05-statistically significant difference.

The identical superscript indicates statistically significant difference amongst designated groups for respective linear measurement (eg. A-D, B-E, and C-F).

Quantification of artifacts of CBCT in the presence of implants or prostheses showed that with an increase in the number of implants or prostheses, the mean grayscale values increased. The highest mean grayscale value was seen in full-arch prostheses (170 ± 90.90), while the lowest grayscale value was seen in the single implant with prosthesis (98.03 ± 84.47) (Table 4). The mean grayscale change was greatest in the vicinity of the implant and implant prosthesis, with the greatest (64.64 78.097) at 3 mm and the least (8.63 81.269) at 10 mm (Table 5).

**Table 4:** Comparison of grayscale values of the test group.

Groups	Mean ± SD	TG1	TG2	TG3	TG4	TG5	TG6	TG7
		Post Hoc p-values						
Control	86.33 ± 66.8	0.426	1.000	0.000	0.000	0.000	0.000	0.000
TG1	101.41 ± 84.1	1						
TG 2	98.03 ± 84.47	1.000	1					
TG3	119.68 ± 89.06	0.092	0.014	1				
TG4	123.66 ± 92.07	0.010	0.001	1.000	1			
TG5	132.15 ± 85.36	0.000	0.000	1.000	1.000	1		
TG6	152.98 ± 89.68	0.000	0.000	0.000	0.000	0.000	1	
TG7	170 ± 90.90	0.000	0.000	0.000	0.000	0.000	0.000	1

*p* > 0.05 statistically non-significant difference.

*p* < 0.05-statistically significant difference.

**Table 5:** Mean grayscale change at various distances from the center of implant: a comparison using one-way ANOVA followed by post-hoc Tukey HSD test.

Distance (mm)	Mean grayscale	Standard deviation	Standard error	p-value
3 mm	64.64^abc^	78.097	3.013	0.000*
5 mm	51.55^ade^	75.977	2.931	
7.5 mm	25.17^bdf^	79.199	3.055	
1 0 mm	8.63^cef^	81.269	3.135	

− ∆G = G_*TG*_ − G_*C1*_*.*

*p* > 0.05 statistically non-significant difference.

* -p < 0.05-statistically significant difference.

The canine and molar implant artifacts followed geometric distribution patterns. The grayscale changes at a 3 mm distance from the center of the canine implant (1 mm from the surface) had the highest values at 90° (94.33 ± 38.43) (lingual) and 270° (98.62 ± 41.722) buccally. Similarly, the highest grayscale values were observed around the molar implant at 90° (87.33 ± 49.808) and 270° (122.19 ± 74.682). In the region between canine and molar, reduced grayscale values were observed, i.e., at canine 180° (− 17.14 ± 62.041) and molar 0° (− 26.10 ± 61.173) due to the influence of implant or prosthesis (Table 6).

**Table 6:** Mean grayscale change at 3 mm distance at various angular regions of interest: a one-way ANOVA comparison followed by post-hoc Tukey HSD test.

3 mm	Angular measurement	Mean	Standard deviation	Std. error	F	Significance
Canine	0	32.60^abcdef^	49.436	11.054	16.499	0.000*
Canine	65	73.81^ag^	39.615	8.645		
Canine	90	94.33^bh^	38.435	8.387		
Canine	115	81.19^ci^	37.237	8.126		
Canine	180	− 17.14^ghijkl^	62.041	13.539		
Canine	245	76.62^dj^	35.754	7.802		
Canine	270	98.62^ek^	41.722	9.105		
Canine	295	86.67^fl^	42.499	9.274		
Molar	0	− 26.10^abcdef^	61.173	13.349	13.292	0.000*
Molar	65	62.10^a^	55.994	12.219		
Molar	90	87.33^bh^	49.808	10.869		
Molar	115	59.71^cgi^	56.162	12.255		
Molar	180	− 9.10^dfghijkl^	81.392	17.761		
Molar	245	99.29^el^	70.978	15.489		
Molar	270	122.19^fj^	74.682	16.297		
Molar	295	74.05^ k^	60.918	13.293		

*p* > 0.05 statistically non-significant difference.

* -p < 0.05-statistically significant difference.

The identical superscript indicates statistically significant difference amongst designated groups for canine and molar.

The grayscale changes at a 5 mm distance from the center of the canine implant had the highest values at 90° and 270°. The highest grayscale values were observed around the molar implant at 115° and 270° (Table 7).

**Table 7:** Mean grayscale change at 5 mm distance at various angular regions of interest: A one-way ANOVA comparison followed by post-hoc Tukey HSD test.

5 mm	Angular measurement	Mean	Standard deviation	Std. error	F	P-value
Canine	0	27.38^ab^	35.032	7.645	8.671	0.000*
Canine	65	60.00^c^	55.987	12.217		
Canine	90	68.62^d^	66.211	14.449		
Canine	115	27.76	55.866	12.191		
Canine	180	− 20.38^cdef^	60.955	13.302		
Canine	245	58.05	65.218	14.232		
Canine	270	79.33^ae^	31.696	6.917		
Canine	295	79.14^bf^	40.768	8.896		
Molar	0	− 10.52^abcde^	80.257	17.514	11.569	0.000*
Molar	65	28.52	32.331	7.055		
Molar	90	61.90^a^	35.148	7.670		
Molar	115	70.19^bf^	42.621	9.301		
Molar	180	− 14.90f.	48.884	10.667		
Molar	245	65.57^cg^	53.784	11.737		
Molar	270	76.10^d^	48.131	10.503		
Molar	295	68.33^eg^	46.167	10.075		

*p* > 0.05 statistically non-significant difference.

* -p < 0.05-statistically significant difference.

The identical superscript indicates statistically significant difference amongst designated groups for canine and molar.

The grayscale changes at a 7.5 mm distance from the center of the canine implant had the highest values at 115° and 270°. The highest grayscale values were observed around the molar implant at 90° and 270° (Table 8).

**Table 8:** Mean grayscale change at 7.5 mm distance at various angular regions of interest: A comparison using one-way ANOVA followed by post-hoc Tukey HSD test.

7.5 mm	Angular measurement	Mean	Standard deviation	Std. error	F	Sig.
Canine	0	31.10	50.773	11.080	1.464	0.184
Canine	65	31.86	55.408	12.091		
Canine	90	34.90	56.508	12.331		
Canine	115	42.86	69.749	15.220		
Canine	180	− 5.86	56.652	12.362		
Canine	245	42.76	72.139	15.742		
Canine	270	47.48	72.265	15.769		
Canine	295	25.05	69.168	15.094		
Molar	0	− 15.67^ad^	50.019	10.915	4.894	0.000*
Molar	65	33.71^a^	73.382	16.013		
Molar	90	50.48	83.572	18.237		
Molar	115	46.10	58.944	12.863		
Molar	180	− 21.48^bcde^	50.826	11.091		
Molar	245	46.24	58.761	12.823		
Molar	270	59.52^c^	75.221	16.415		
Molar	295	50.19^e^	63.952	13.955		

*p* > 0.05 statistically non-significant difference.

* -p < 0.05-statistically significant difference.

The identical superscript indicates statistically significant difference amongst designated groups for molar.

The grayscale changes at a 10 mm distance from the center of the canine implant had the highest values at 90° and 270°. The highest grayscale values were observed around the molar implant at 90° and 270° (Table 9).

**Table 9:** Mean grayscale change at 10 mm distance at various angular regions of interest: a one-way ANOVA comparison followed by post-hoc Tukey HSD test.

10 mm	Angular measurement	Mean	Standard deviation	Std. error	F	Sig.
Canine	0	16.33	33.900	7.398	8.786	0.000*
Canine	65	24.71	53.446	11.663		
Canine	90	32.81	53.479	11.670		
Canine	115	20.48	58.644	12.797		
Canine	180	− 29.24^abc^	56.235	12.272		
Canine	245	59.52^a^	90.882	19.832		
Canine	270	65.81^b^	89.932	19.625		
Canine	295	54.90^c^	85.537	18.666		
Molar	0	− 7.86	53.448	11.663	16.499	0.000*
Molar	65	37.76	74.759	16.314		
Molar	90	44.38	73.354	16.007		
Molar	115	17.00	66.799	14.577		
Molar	180	− 8.14	46.654	10.181		
Molar	245	17.43	107.206	23.394		
Molar	270	50.29	72.613	15.845		
Molar	295	45.71	81.668	17.821		

*p* > 0.05 statistically non-significant difference.

*-p < 0.05-statistically significant difference.

The identical superscript indicates statistically significant difference amongst designated groups for canine.

In the region between canine and molar, reduced grayscale values were observed, i.e., at canine 180° and molar 0° due to the influence of implant or prosthesis (Tables 6, 7, 8, 9).

Grayscale and change in grayscale(∆G) were evaluated at 0.25 mm,10 mm, and 20 mm Voxel integration scale (VIS). The grayscale value increased at a VIS (10 mm and 20 mm), and statistically significant (p < 0.01) results were observed in mean grayscale and grayscale change (∆G) at different VIS (Tables 10 and 11).

**Table 10:** Mean grayscale at different voxel integration scales (VIS): a one-way ANOVA comparison followed by post-hoc Tukey HSD test.

Groups	Mean	Standard deviation	p-value
0.25 mm	83.79^a^	80.86	0.000*
10 mm	134.20^a^	89.50	
20 mm	151.33^a^	85.33	

The identical superscript indicates statistically significant difference amongst designated groups.(*-p<0.05-statistically significant difference)

**Table 11:** Mean grayscale change(∆G) at different integration scales (VIS): A one-way ANOVA comparison followed by post-hoc Tukey HSD test.

Groups	Mean	Standard deviation	Standard error	F	p-value
0.25 mm	31.26^a^	81.507	2.723	5.450	0.004*
10 mm	37.27	81.892	2.736		
20 mm	43.96^a^	81.026	2.707		

− ∆G = G_*TG*_ − G_*C1*_*.*

*p* > 0.05 statistically non-significant difference.

*-p < 0.05-statistically significant difference.

The identical superscript indicates statistically significant difference amongst designated groups.

## Discussion

The increasing dental awareness has led to increased implant-supported prostheses in partially and completely edentulous patients. The presence of dental implants and prostheses causes artifacts such as beam hardening, scattering, etc. These artifacts also reduce imaging diagnostic accuracy, making diagnosing peri-implant features difficult^17^. CBCT is a reliable radiographic tool with accuracy in the 0.163 mm to 0.40 mm range, as shown in our study. The results are supported by Maroua AL^18^, Ganguly R^19^, and Menezes et al.^20^.

As we progressed from the single implant (TG-1) to full-arch implant-supported prosthesis (TG-7), the dimensional accuracy was reduced, confirming the co-relation between metal implants or implant-supported prostheses and the accuracy of CBCT image. The highly significant difference in observations can be explained by the role of artifacts induced by prostheses. The present study's findings prove that the artifacts increase as metal in implants and prostheses increases. An accurate delineation of buccal and lingual cortical plates in the peri-implant or prostheses area becomes difficult. The implant or prostheses produced artifacts strong enough to cause a dimensional error. This can be explained by the direction of the implant artifacts, which are diagonal to the implant axis, causing in-accuracies in transverse and sagittal planes making radiographic bone appearance wider than reality. This result is supported by Grobe A^21^, who, in an in-vivo study after placement of multiple implants, found a statistically highly significant (*p* value 0.003) difference between transverse measurements in CBCT and histological sections with a mean overestimation of 0.3 ± 0.04 mm in the CBCT image.

The TG RLM at the inferior axial plane showed no significant dimensional observation compared to C0. This can be explained by the reduced influence of prostheses as distance increases (at the inferior border). Studies have shown a sub-millimeter difference in CBCT measurements compared to the gold standard (caliper measurements)^11,22,23–25^. The present study evaluated and compared measurements in all three planes, endorsing the conclusion that CBCT is a reliable diagnostic tool for linear measurements. However, previous studies focused on one or two implant configurations across multiple models, but none looked at the impact of prostheses on accuracy. The present study found that as we progressed from TG1 to TG 7, the dimensional accuracy of CBCT was reduced. As metals in implants and prostheses increase, accurate delineation of buccal and lingual cortical plates in the peri-implant and implant-supported prostheses area becomes difficult. Also, the artifacts increase in a direction diagonal to the implant axis, causing accuracies in transverse and sagittal planes making bone appear wider than reality, causing the dimensional error^21^.

Beam hardening is the most significant metal-induced artifact of CBCT, causing grayscale change^8,26^. This is because lower energy X-ray beams from polychromatic X-rays are scattered and absorbed by denser metal and even lighter titanium. In comparison, higher energy beams pass through the surface/body of the metal object and are recorded as a high-energy beam by the detector^8,27^. Beam hardening causes metallic structure image distortion by differential absorption, known as a cupping artifact.

The lowest greyscale values observed in TG2 can be explained by the cupping effect, which is less with an 'only' implant (TG1) than for an implant with a prosthesis (TG2) (Table 10). The current study showed differential absorption of x-rays because of the metallic structure resulting in decreased grayscale values at the immediate periphery, as also shown by a study by Kou^28^, who found the least grayscale value change in the proximity of a single dental implant prosthesis. The single implant with prostheses showed a higher decrease in grayscale values in the periphery than the only implant due to the additional cupping effect of increased metal content. Because of the artifact additive effect, the grayscale values increased with the number of implants^8,28^.

Grobee A^21^ supports Benic GI^9^ fontanelle^29^, who found significant artifacts up to 3.5 cm from the implant. The artifacts were present both in proximity and away from implants. The current results have emphasized the change in grayscale, with the diminishing influence of metal as the distance from the implant or prostheses increases.

The pattern of artifacts around the implant was observed at 3 mm, 5 mm, 7.5 mm, and 10 mm from the center of the implant, which showed a higher standard deviation at 0° and 180° in both the canine and molar region at 3 mm distance, indicating a greater amount of artifacts variation and worse image quality at these angular regions of interest (ROI)^30^. Schulze^8^, Benic^9^, and Pauwels^31^ also found a similar decrease in grayscale values at the interproximal region between two implants or prostheses. Benic^9^ recorded the greatest grayscale change at points perpendicular to the mandibular axis, corroborating with the present study. While Benic^9^ found reduced grayscale values in mesial and distal regions of a single implant, the present study recorded negative grayscale changes in the interproximal area and distal to molar region, but not at 0° (mesial) in the canine region where only reduced grayscale change values were recorded. Also, a study by Pauwels R^31^ concluded that the area between two metal objects was most affected by artifacts, which concurs with the present study.

The mean grayscale change at the superior axial plane was higher (50.72) than at the inferior axial plane (33.36), indicating a greater influence of implant prosthesis. Likewise, the grayscale value increased at a higher VIS with significant observations in mean grayscale values and changes in grayscale values as a VIS indicator accuracy is proportional to VIS. The findings endorse previous observations of Candemill Ap^32^, who concluded that smaller voxel sizes were preferred for higher accuracy.

The present study revealed higher but statistically non-significant grayscale values when the canine region (126.04 ± 93.26) was compared with the molar region (120.17 ± 86.48). The results indicated that artifacts produced are independent of the area or place of implant or prosthesis placement. This result concurs with earlier studies by Benic GI^9^ and Fontenele^29^, who also did not find any correlation between the position of the implant and artifacts.

The Limitation of the present study is a simplified in-vitro situation where the role of soft tissue around the mandible is eliminated. Since the study was carried out on a dry mandible, the reported results should be considered optimal, as precision and reliability are most likely lowered in clinical settings because of factors such as patient movement, adjoining soft tissue, and other intra-oral factors such as restorations that may influence linear measurements in a CBCT scan. Furthermore, lower VIS should be used to reduce artifacts and improve accuracy, as higher VIS increases artifacts. Moreover, the influence of different CBCT equipment/software for image reconstruction could also be studied.

## Conclusion

The study aimed to assess the accuracy of CBCT as a measuring tool and quantify metal artifacts produced by CBCT of the mandible in the presence of implants and implant-supported prostheses. The results showed that the presence of implants and prostheses can cause artifacts, such as beam hardening and scattering, which can reduce the accuracy of the images. These artifacts were found to increase with the number of implants and the size of the prostheses. The artifacts were more pronounced in the presence of more metal, such as in full-arch implant-supported prostheses. The study also found that the artifacts were more pronounced in the superior axial plane than in the inferior axial plane. Furthermore, lower VIS should be used to reduce artifacts and improve accuracy, as higher VIS increases artifacts. Therefore, caution should be exercised when interpreting CBCT images with implants and prostheses, and additional diagnostic imaging modalities may be necessary to obtain more accurate information. Overall, this study provides important insights into the use of CBCT in implant dentistry and highlights the need for further research to improve imaging quality and reduce artifacts in CBCT images.

### Supplementary Information

Supplementary Information.

## Data Availability

All data generated or analysed during this study are included in its supplementary information files.

## References

[1] Danforth RA (2003). Cone beam volume tomography: A new digital imaging option for dentistry. J. Calif. Dent. Assoc..

[2] Shah N, Bansal N, Logani A (2014). Recent advances in imaging technologies in dentistry. World J. Radiol..

[3] Hendrikx AW, Maal T, Dieleman F, Van Cann EM, Merkx MA (2010). Cone-beam CT in the assessment of mandibular invasion by oral squamous cell carcinoma: Results of the preliminary study. Int. J. Oral Maxillofac. Surg..

[4] Closmann JJ, Schmidt BL (2007). The use of cone beam computed tomography as an aid in evaluating and treatment planning for mandibular cancer. J. Oral Maxillofac. Surg..

[5] Tyndall DA, Rathore S (2008). Cone-beam CT diagnostic applications: Caries, periodontal bone assessment, and endodontic applications. Dent. Clin. N. Am..

[6] Mozzo P, Procacci C, Tacconi A, Martini PT, Andreis IA (1998). A new volumetric CT machine for dental imaging based on the cone-beam technique: Preliminary results. Eur. Radiol..

[7] Scarfe WC (2005). Imaging of maxillofacial trauma: Evolutions and emerging revolutions. Oral Surg. Oral Med. Oral Pathol. Oral Radiol. Endod..

[8] Schulze R, Heil U, Gross D, Bruellmann DD, Dranischnikow E, Schwanecke U, Schoemer E (2011). Artefacts in CBCT: A review. Dentomaxillofac. Radiol..

[9] Benic GI, Sancho-Puchades M, Jung RE, Deyhle H, Hämmerle CH (2013). In vitro assessment of artifacts induced by titanium dental implants in cone beam computed tomography. Clin. Oral Implants Res..

[10] Lee MY, Song KH, Lee JW, Choe BY, Suh TS (2019). Metal artifacts with dental implants: Evaluation using a dedicated CT/MR oral phantom with registration of the CT and MR images. Sci. Rep..

[11] Torres MG, Campos PS, Segundo NP, Navarro M, Crusoé-Rebello I (2012). Accuracy of linear measurements in cone beam computed tomography with different voxel sizes. Implant Dent..

[12] Sheikhi M, Dakhil-Alian M, Bahreinian Z (2015). Accuracy and reliability of linear measurements using tangential projection and cone beam computed tomography. Dent. Res. J..

[13] Al-Ekrish AA (2021). Comparative study of the accuracy of CBCT implant site measurements using different software programs. Saudi Dent. J..

[14] Amarnath GS, Kumar U, Hilal M, Muddugangadhar BC, Anshuraj K, Shruthi CS (2015). Comparison of cone beam computed tomography, orthopantomography with direct ridge mapping for pre-surgical planning to place implants in cadaveric mandibles: An ex-vivo study. J. Int. Oral Health.

[15] Draenert FG, Coppenrath E, Herzog P, Müller S, Mueller-Lisse UG (2007). Beam hardening artefacts occur in dental implant scans with the NewTom cone beam CT but not with the dental 4-row multidetector CT. Dentomaxillofac. Radiol..

[16] Chen LC, Lundgren T, Hallström H, Cherel F (2008). Comparison of different methods of assessing alveolar ridge dimensions prior to dental implant placement. J. Periodontol..

[17] Schulze RK, Berndt D, d'Hoedt B (2010). On cone-beam computed tomography artifacts induced by titanium implants. Clin. Oral Implants Res..

[18] Maroua AL, Ajaj M, Hajeer MY (2016). The accuracy and reproducibility of linear measurements made on CBCT-derived digital models. J. Contemp. Dent. Pract..

[19] Ganguly R, Ramesh A, Pagni S (2016). The accuracy of linear measurements of maxillary and mandibular edentulous sites in cone-beam computed tomography images with different fields of view and voxel sizes under simulated clinical conditions. Imaging Sci. Dent..

[20] Menezes CC, Janson G, da Silveira MC, Cambiaghi L, Garib DG (2016). Precision, reproducibility, and accuracy of bone crest level measurements of CBCT cross sections using different resolutions. Angle Orthod..

[21] Gröbe A, Semmusch J, Schöllchen M, Hanken H, Hahn M, Eichhorn W (2017). Accuracy of bone measurements in the vicinity of titanium implants in CBCT data sets: A comparison of radiological and histological findings in minipigs. Biomed. Res. Int..

[22] Bohner, LOL., Tortamano, P., Marotti, J. Accuracy of linear measurements around dental implants by means of cone beam computed tomography with different exposure parameters. *Dentomaxillofac Radiol.* **46**(5), 20160377 (2017). 10.1259/dmfr.20160377 PMC5595033 28267928

[23] Rokn AR, Hashemi K, Akbari S, Kharazifard MJ, Barikani H, Panjnoosh M (2016). Accuracy of linear measurements using cone beam computed tomography in comparison with clinical measurements. J. Dent..

[24] Gerlach NL, Meijer GJ, Maal TJ, Mulder J, Rangel FA, Borstlap WA, Bergé SJ (2010). Reproducibility of 3 different tracing methods based on cone beam computed tomography in determining the anatomical position of the mandibular canal. J. Oral Maxillofac. Surg..

[25] Luangchana P, Pornprasertsuk-Damrongsri S, Kiattavorncharoen S, Jirajariyavej B (2015). Accuracy of linear measurements using cone beam computed tomography and panoramic radiography in dental implant treatment planning. Int. J. Oral Maxillofac. Implants.

[26] De Man B, Nuyts J, Dupont P, Marchal G, Suetens P (1999). Metal streak artifacts in X-ray computed tomography: A simulation study. IEEE Trans. Nucl. Sci..

[27] Miracle AC, Mukherji SK (2009). Conebeam CT of the head and neck, part 1: Physical principles. AJNR Am. J. Neuroradiol..

[28] Kuo RF, Fang KM, Ty W, Hu CY (2016). Quantification of dental prostheses on cone-beam CT images by the Taguchi method. J. Appl. Clin. Med. Phys..

[29] Fontenele RC, Nascimento EH, Vasconcelos TV, Noujeim M, Freitas DQ (2018). Magnitude of cone beam CT image artifacts related to zirconium and titanium implants: Impact on image quality. Dentomaxillofac. Radiol..

[30] Terrabuio BR, Carvalho CG, Peralta-Mamani M, Santos PSDS, Rubira-Bullen IRF, Rubira CMF (2021). Cone-beam computed tomography artifacts in the presence of dental implants and associated factors: An integrative review. Imaging Sci. Dent..

[31] Pauwels R, Stamatakis H, Bosmans H, Bogaerts R, Jacobs R, Horner K, Tsiklakis K, SEDENTEXCT Project Consortium (2013). Quantification of metal artifacts on cone beam computed tomography images. Clin. Oral Implants Res..

[32] Candemil AP, Salmon B, Ambrosano GMB, Freitas DQ, Haiter-Neto F, Oliveira ML (2021). Influence of voxel size on cone beam computed tomography artifacts arising from the exomass. Oral Surg. Oral Med. Oral Pathol. Oral Radiol..

